# Antisense Transcripts and Antisense Protein: A New Perspective on Human Immunodeficiency Virus Type 1

**DOI:** 10.3389/fmicb.2020.625941

**Published:** 2021-01-12

**Authors:** Juliette Savoret, Jean-Michel Mesnard, Antoine Gross, Nathalie Chazal

**Affiliations:** Institut de Recherche en Infectiologie de Montpellier (IRIM), CNRS, Université de Montpellier, Montpellier, France

**Keywords:** HIV-1, antisense transcripts, antisense protein, immune response, evolution

## Abstract

It was first predicted in 1988 that there may be an Open Reading Frame (ORF) on the negative strand of the Human Immunodeficiency Virus type 1 (HIV-1) genome that could encode a protein named AntiSense Protein (ASP). In spite of some controversy, reports began to emerge some years later describing the detection of HIV-1 antisense transcripts, the presence of ASP in transfected and infected cells, and the existence of an immune response targeting ASP. Recently, it was established that the *asp* gene is exclusively conserved within the pandemic group M of HIV-1. In this review, we summarize the latest findings on HIV-1 antisense transcripts and ASP, and we discuss their potential functions in HIV-1 infection together with the role played by antisense transcripts and ASPs in some other viruses. Finally, we suggest pathways raised by the study of antisense transcripts and ASPs that may warrant exploration in the future.

## Introduction

**Figure 1:**
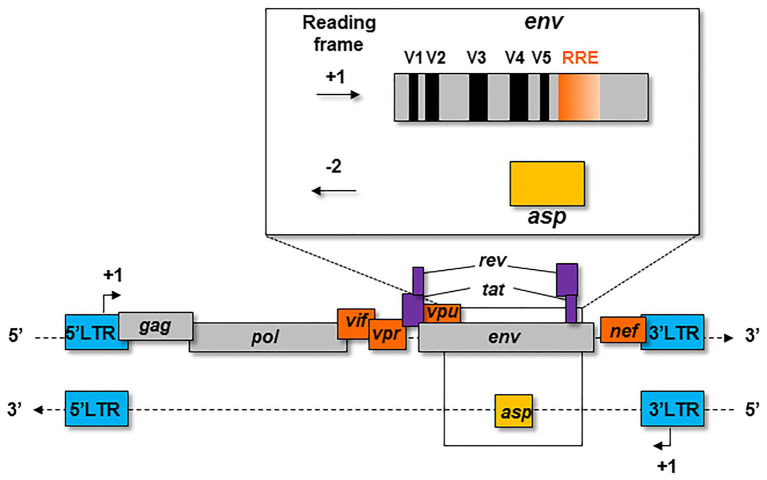
Schematic representation of the *asp* gene within the proviral genome of HIV-1. The *asp* gene overlaps the *env* gene in the −2 frame. The *asp* gene overlaps the hypervariable regions V4 and V5 of *env* and partly overlaps the Rev Responsive Element (RRE).

The first hypothesis on the existence of the *asp* gene overlapping *env* in the -2 frame on the antisense strand of HIV-1 proviral genome was formulated in 1988 (Miller, 1988; Figure 1). At that time, this postulate had little impact on the retrovirology research community, and the *bona fide* existence of this gene was highly contested for several years. The discovery of an Open Reading Frame (ORF) on the negative strand of the HIV-1 genome was not in agreement with the generally-accepted retrovirology dogma, stipulating that retroviral genes are only expressed from a unique promoter located in the 5' Long Terminal Repeat (LTR; Figure 1).

Despite early skepticism and the lack of specific tools to selectively identify rare antisense transcripts and detect a strongly hydrophobic and “young” protein like AntiSense protein (ASP), several potential antisense ORFs and (ASPs) were described for different Retroviruses. One ORF was found on the antisense strand of the human T-cell leukemia virus type 1 (HTLV-1) genome, and antisense transcripts were detected in HTLV-1 infected T-cells (Larocca et al., 1989; Barbeau and Mesnard, 2015; Matsuoka and Mesnard, 2020). An ORF located on the complementary DNA strand of the Feline Immunodeficiency Virus (FIV) envelope gene was also identified (Briquet et al., 2001). Although antisense transcripts were detected in FIV-infected cell lines and in tissues of infected cats, their coding capacity has not been demonstrated yet (Briquet et al., 2001). The first retroviral ASP formally identified was the basic leucine zipper factor (bZIP) of HTLV-1 (Gaudray et al., 2002), followed by the identification of the ASPs of HTLV-2, HTLV-3, and HTLV-4 (Halin et al., 2009; Larocque et al., 2011). More recently, an antisense gene was characterized in the genome of the Simian T-Leukemia Virus type 1 (STLV-1), and antisense transcripts were characterized in STLV-1-infected cells (Miura et al., 2013). This gene encodes a protein *in vitro* which displayed functions similar to that of HBZ (Miura et al., 2013). Antisense transcripts were also detected in Murine Leukemia Virus (MLV; Rasmussen et al., 2010), Bovine Immunodeficiency Virus (BIV; Liu et al., 2015), and Bovine Leukemia Virus (BLV; Durkin et al., 2016). However, no ASPs associated with these transcripts have thus far been identified.

The presence of antisense transcripts was first observed in an HIV-1-infected cell line in 1990 (Bukrinsky and Etkin, 1990), and ASP itself was first detected in 1995 (Vanhée-Brossollet et al., 1995). Despite this promising discovery, very few studies were published on the investigation of ASP and its potential antisense transcripts. Several HIV-1 *in vitro* antisense transcripts were described in transfected and infected cell lines (Michael et al., 1994; Landry et al., 2007; Kobayashi-Ishihara et al., 2012; Saayman et al., 2014), and two studies recently detected antisense transcripts in CD4^+^ T cells of infected patients (Zapata et al., 2017; Mancarella et al., 2019). In 2015, two reports were published demonstrating the presence of CD8^+^ T cells directed against several ASP peptides in HIV-1-infected patients (Berger et al., 2015; Bet et al., 2015). Recently, the presence of ASP-specific antibodies was detected in the plasma of HIV-1-infected individuals (Savoret et al., 2020), thereby confirming the pioneer study of Vanhée-Brossollet et al. which first proposed the existence of ASP-specific antibodies (Vanhée-Brossollet et al., 1995), and further suggesting that ASP is expressed and immunogenic *in vivo*.

## Origin, Evolution, and Conservation of the *ASP* Gene

**Figure 2:**
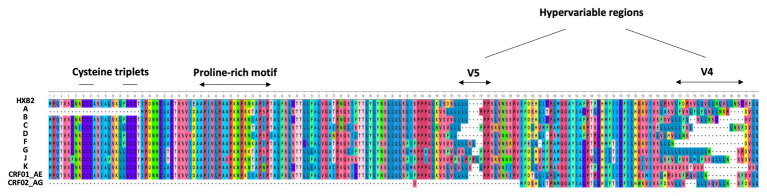
Alignment of the consensus amino acid sequence of AntiSense Protein (ASP) encoded by the major subtypes and circulating recombinant forms (CRFs) of HIV-1 (sequences were retrieved from HIV-1 data bases, Los Alamos National Laboratory and analyzed using Unipro UGENE: a unified bioinformatics tollkit” Okonechnikov; Golosova; Fursov. Bioinfomatics 2012 28: 1166–1,167). The first line represents the reference HXB2 sequence of ASP. The main motifs of ASP are indicated.

In 2016, Cassan et al. developed a new approach to characterize the origin, conservation, and evolution of the *asp* gene within the four phylogenetic groups of HIV-1 (M, N, O, and P; Cassan et al., 2016). As *asp* overlaps the *env* gene in the −2 frame (Figure 1), the ASP ORF could not be characterized with classical bioinformatics tools based on the measurement of selection pressures on a DNA fragment. To overcome this difficulty, Cassan et al. (2016) considered the appearance of start and stop codons in the −2 frame of the *env* gene. The *ASP* ORF was detected in sequences of the most prevalent HIV-1 subtypes and circulating recombinant forms (CRFs) of the group M, while it was not observed in sequences from the endemic O group or in the rare N and P groups (Cassan et al., 2016). These results indicated that the creation of *asp* was concomitant with the emergence of the group M in humans. It is noteworthy that the A subtype and its recombinant forms display a stop codon at the beginning of the ASP ORF that is followed in more than 90% of the sequences by a start codon which maintains the ASP ORF (Cassan et al., 2016). As a result, the subtype A and its recombinant forms encode a shorter version of ASP devoid of the first 25 residues (Figure 2), including the two cysteine triplets of ASP which have been shown to be involved in ASP multimerization in transfected cells *in vitro* (Torresilla et al., 2013; Liu et al., 2019).

Despite the high degree of conservation of *asp* within the M group (Table 1; Figure 2), 16% of the sequences from the A, B, C, and G subtypes and from the CRF01_AE did not display the ASP ORF. This observation, in conjunction with *de novo* creation of *asp*, strongly suggests that *asp* is an auxiliary gene which can be lost without compromising the virion structure or viral replication. However, this would not mean that the product of this gene, ASP, is dispensable *in vivo*. Indeed, most *de novo* created translational products of viral genes play an important role in viral pathogenicity or spread (Li and Ding, 2006). Computer simulation showed that the high degree of ASP ORF conservation within the pandemic group M was unlikely to be accidental (Cassan et al., 2016). This finding, together with the study of *asp* sequences of the A subtype and its recombinants, provided evidence of a selection pressure acting to maintain *asp* in the group M and strongly suggested that *asp* is a *bona fide* gene.

**Table 1:** Fraction of the sequences displaying the AntiSense Protein Open Reading Frame (length >150 codons) within the main subtypes and circulating recombinant forms (CRFs) of HIV-1 group M (Cassan et al., 2016).

HIV-1 group M subtypes	% of sequences with ASP ORF
A	74
B	85
C	84
D	50
F	32
G	88
H	0
J	50
K	50
CRF01_AE	89
CRF02_AG	7

Altogether, this study strongly suggests that the *asp* gene which appeared concomitantly with, and is uniquely conserved within, the HIV-1 pandemic group M, is the 10th HIV-1 gene and that its transcriptional and/or translational products may endow the virus with an evolutionary advantage (Cassan et al., 2016). Of note, 1 year after this study, using an extensive sequence analysis (660 viral strains), another study found that *asp* mutations were associated with mutations of the hypervariable region V3 of *env* and thereby proposed that *asp* mutations could be linked to viral tropism and different co-receptor usage (Dimonte, 2017).

## The Antisense Transcriptional Activity of HIV-1

The antisense transcriptional activity of HIV-1 is initiated from a promoter located in the 3' LTR. As a consequence of HIV-1 LTR bidirectionality, initiation of HIV-1 sense and antisense transcriptions depends the on binding of common transcription factors, mainly NF-kB and SP1 (Michael et al., 1994; Vanhée-Brossollet et al., 1995; Peeters et al., 1996; Landry et al., 2007; Kobayashi-Ishihara et al., 2012; Arpin-André et al., 2014). As described in eukaryotic cells (Adhya and Gottesman, 1982; Callen et al., 2004; Shearwin et al., 2005), the concomitant initiation of retroviral sense and antisense transcriptional activities can possibly induce transcriptional interference. In order to prevent transcriptional interference, HIV-1 sense and antisense transcriptions might function antagonistically, similar to what has been described for HTLV-1 (Barbeau and Mesnard, 2015). Several data support this hypothesis. Indeed, in productively HIV-1-infected-cells, sense transcription has been shown to predominate over antisense transcription, in agreement with the preferential activation of HIV-1 sense transcription by Tat (Michael et al., 1994; Vanhée-Brossollet et al., 1995; Landry et al., 2007; Kobayashi-Ishihara et al., 2012; Laverdure et al., 2012; Arpin-André et al., 2014). Moreover, HIV-1 antisense transcriptional activity was shown to increase upon 5' LTR deletion *in vitro* (Klaver and Berkhout, 1994; Landry et al., 2007), and the ratio of HIV-1 sense/antisense transcriptions was found to be 10-fold higher in activated CD4^+^ T cells than in resting cells such as monocyte-derived macrophages and dendritic cells (Laverdure et al., 2012). Finally, a study recently reported the expression of antisense transcripts in T cells latently infected *in vitro* with a reporter virus, further suggesting that HIV-1 antisense expression can occur when sense transcription is low (Kobayashi-Ishihara et al., 2018).

### The Antisense Transcripts of HIV-1 *in vitro*

**Figure 3:**
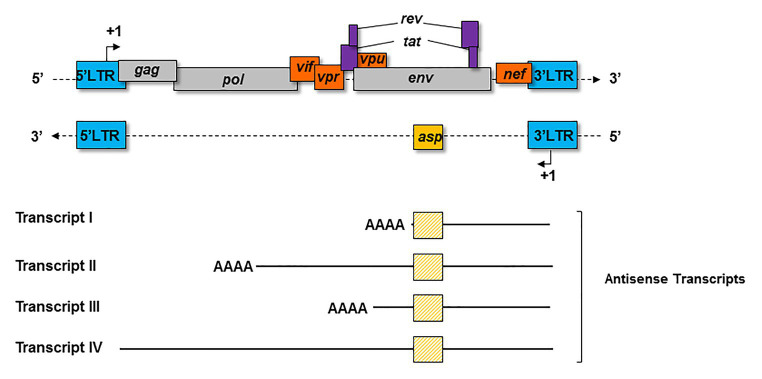
Schematic representation of the principal antisense transcripts that were described *in vitro* (transcripts I to IV). The poly-A tails and the ASP ORF (yellow square) are represented for each transcript.

Four major kinds of antisense transcripts were characterized so far *in vitro* (Michael et al., 1994; Landry et al., 2007; Kobayashi-Ishihara et al., 2012; Saayman et al., 2014). A transcript of 2.3 Kb (Transcript I; Figure 3) was first detected in HIV-1-infected cell lines (Michael et al., 1994). Using a strand-specific RT-PCR as previously described (Barbeau and Mesnard, 2015), a 5 Kb transcript (Transcript II; Figure 3) initiating at several transcription starting sites (TSS) was subsequently characterized in transfected HEK 293 T cells (Landry et al., 2007). Using the same technique, a third antisense transcript of 3 Kb (Transcript III; Figure 3) was detected in HIV-1-infected MAGIC-5 cells and in several chronically infected cell lines (Kobayashi-Ishihara et al., 2012). Transcript III promotes the initiation and maintenance of viral latency by recruiting the Polycomb Repressor Complex 2 (PRC2) to the 5′ LTR of HIV-1 (Zapata et al., 2017). A role in the maintenance of viral latency was also reported for a genome-length antisense transcript devoid of poly-A tail (Transcript IV; Figure 3), which was detected in two chronically infected T-cell lines (Saayman et al., 2014). In these cells, Transcript IV behaved as a long non-coding (lnc) RNA by recruiting chromatin modifying enzymes, such as DNA methyltransferase 3a (DNMT3a), Enhancer of Zeste Homolog 2 (EZH2), and Histone Deacetylase (HDAC)-1, to the 5' LTR of the provirus (Saayman et al., 2014; Lange et al., 2020). This multiplicity of antisense transcripts characterized *in vitro* may be explained by the use of different methodological approaches in different studies. However, it is also plausible that HIV-1-infected cells express several types of antisense transcripts during the retroviral cycle.

As we saw above, a number of publications suggested that HIV-1 antisense transcripts are preferentially expressed in cells displaying low levels of sense transcripts (Vanhée-Brossollet et al., 1995; Landry et al., 2007; Kobayashi-Ishihara et al., 2012; Laverdure et al., 2012; Arpin-André et al., 2014; Saayman et al., 2014; Barbeau and Mesnard, 2015). Indeed, Transcript IV was detected in latently HIV-1-infected cell lines (Saayman et al., 2014). Antisense transcripts were also found in CEM T cells that were latently infected with a VSVg-pseudotyped HIV NL-E_ΔEnv_ virus that carries a EGFP reporter gene (Kobayashi-Ishihara et al., 2018). In this study, infected cells were sorted by flow cytometry to obtain a cell population composed of latently HIV-1-infected cells (EGFP-negative; Kobayashi-Ishihara et al., 2018). Moreover, it was proposed that the HIV-1 antisense transcripts may interfere with virus reactivation from latency as the addition of latency reversal agents only reactivated sense transcription in latently infected cells lacking antisense transcripts (Kobayashi-Ishihara et al., 2018). Altogether, this study further suggests that HIV-1 antisense transcription might be involved in the maintenance of latency.

Antisense transcription may also potentially occur in HIV-1 productively infected cells. Indeed, Transcript III and various antisense transcripts were detected 3 days post-infection, in MAGIC-5 cells (Kobayashi-Ishihara et al., 2012) and H9-infected cells (Bukrinsky and Etkin, 1990), respectively. Finally, Kobayashi-Ishihara et al. (2018) detected antisense transcripts in HIV-1 peripherical blood mononuclear cells (PBMCs) stimulated with phytohaemagglutinin (PHA), even though they were much less abundant than sense transcripts.

### The Antisense Transcripts of HIV-1 *in vivo*

In one study, HIV-1 antisense transcripts were detected in CD4^+^ T cells isolated from five HIV-1-infected patients following CD3/CD28 stimulation but not in unstimulated CD4^+^ T cells (Mancarella et al., 2019), whereas another publication reported antisense transcripts in HIV-1-infected resting CD4^+^ T cells (Zapata et al., 2017). This apparent discrepancy observed in these two studies requires further evaluations. A possible explanation may reside in different RT-qPCR methods used to detect antisense transcripts but also in the small number of patients that was included in these two studies (five and three patients; Zapata et al., 2017; Mancarella et al., 2019). In any case, it cannot be excluded that antisense transcripts are expressed in both productively and latently HIV-1 infected cells *in vivo*. Productively HIV-1 infected cells may indeed co-express antisense mRNAs encoding ASP and antisense transcripts acting as *bona fide* lncRNAs through the recruitment of histone modifying enzymes to the 5' LTR of HIV-1, thereby playing a role in the establishment of viral latency. Once viral latency is established, HIV-1 antisense transcripts may still contribute to the maintenance of latency through the recruitment of enzymes responsible of the silencing of the 5’LTR. As latently HIV-1-infected cells express very low levels of HIV-1 sense transcripts, they might also express antisense mRNAs encoding ASP.

## Structure and Subcellular Localization of ASP

### Structure of ASP

**Figure 4:**
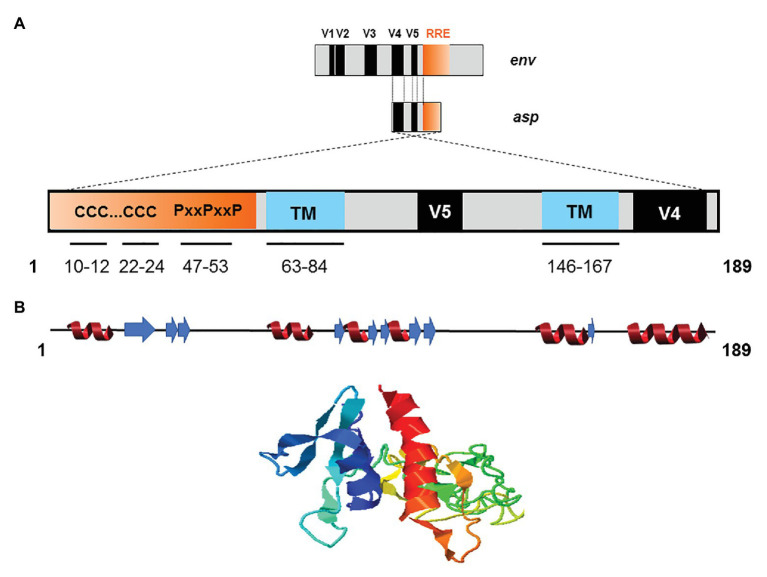
Schematic representation of the primary, secondary, and tertiary structures of ASP. **(A)** Conserved cysteine triplets (CCC), Proline-rich SH3 domain-binding motifs (PxxPxxP), putative transmembrane (TM) domains. The theorical molecular weight of ASP is 20235.26 Da (reference sequence HXB2 using Predict Protein software). Using PONDR VL3, PONDR VSL2, and PONDR VLXT software, it was predicted that, respectively, 9.52, 13.76, and 18.52 of ASP amino acids exist in a disordered state. Only four regions of ASP are expected to exist in disordered states (residues 1–2; 4–4; 44–55; 97–116 predicted using PONDR VSL2 software). **(B)** Predicted secondary (alpha-helix in red and beta-sheet in blue) and tertiary structures of ASP. The reference sequence of *ASP* was submitted to the I-Tasser server (https://zhanglab.ccmb.med.umich.edu/I-TASSER/) to obtain a model *via* a threading prediction. C-Score = −4.07, estimated TM-score = 0.28 ± 0.09, estimated RMSD = 15.0 ± 3.5 Å.

The creation of *de novo* proteins appears to be a significant element in the evolution of viruses (Li and Ding, 2006; Rancurel et al., 2009). Recent *de novo* creation of *asp* gave rise to a translation product which has been named ASP (Miller, 1988; Vanhée-Brossollet et al., 1995; Clerc et al., 2011; Torresilla et al., 2013; Berger et al., 2015; Bet et al., 2015; Affram et al., 2019; Savoret et al., 2020), a small strongly hydrophobic protein consisting of 189 amino acids (reference sequence HXB2). ASP contains 14 conserved cysteine residues, seven of which are located in the N-terminal region (cysteine ^7^C and two cysteine triplets ^10^CCC^12^ and ^22^CCC^24^), two SH3 domain-binding motifs (^47^PXXPXXP^53^), and two strongly hydrophobic putative transmembrane (TM) domains (Figure 4). Most *de novo* created proteins are translated from overlapping genes whose sequence composition is biased toward disorder-promoting amino acids (Rancurel et al., 2009). However, only 9.52, 13.76, and 18.52% of ASP amino acids were predicted to exist in a disordered state according to different software (Figure 4). This relatively low level of disorder could be explained by the strong constraint exerted by overlap with the *env* gene and by structural constraints associated with the Rev. Responsive Element (RRE) sequence (Figure 4).

Due to the overlapping of *env*/*asp* genes, ASP domains are unevenly conserved among the HIV-1 subtypes and CRFs. In fact, the N-terminal extremity of ASP is well conserved except ASP encoded by subtype A and its recombinants, which have shortened N-terminal extremities (Figure 2; Cassan et al., 2016). Conversely, the central region and the C-terminal extremity of ASP, which overlap the hypervariable regions V5 and V4 of *env*, are subjected to strong sequence variations among the different subtypes and CRFs of HIV-1 (Figures 1, 4). ASP was reported to multimerize in mammalian cell lines (COS-7 and HEK 293 T cells) expressing a codon-optimized ASP (Torresilla et al., 2013; Liu et al., 2019). It was recently demonstrated that the capacity of ASP to form aggregates in these cells was mediated by its N-terminal region and, more specifically, by its cysteine residues (Liu et al., 2019). Interestingly, the deletion of the first 15 residues of ASP and the use of a subtype-A ASP reduced the number of multimers detected by Western blot (Liu et al., 2019). As protein aggregates are targeted by constitutive and inducible autophagy (Menzies et al., 2017), it was proposed that ASP multimerization disturbed the autophagic flux in mammalian cell lines, and induced its own degradation by autophagy (Torresilla et al., 2013). Moreover, mammalian cells expressing ASP had more abundant levels of LC3b-II and Beclin-1 than non-ASP expressing cells, and ASP was found to co-immunoprecipitate with LC3-IIb (Torresilla et al., 2013; Liu et al., 2019). Further analyses performed in transfected HEK 293 T cells showed that ASP co-immunoprecipitated with p62, a protein involved in induced autophagy (Klionsky et al., 2016; Liu et al., 2019).

In HEK 293 T cells co-transfected with expression vectors encoding ASP and a His-tagged ubiquitin, Western blot analyses performed following a co-immunoprecipitation using an anti-His antibody strongly suggested that ASP was ubiquitinated (Liu et al., 2019). Although these promising results suggest that autophagy regulates ASP levels in mammalian cells (Torresilla et al., 2013; Liu et al., 2019), they might be taken with caution as both of the above-mentioned studies used transient transfection of eukaryotic expression vector harboring a human codon-optimized ASP cDNA (Torresilla et al., 2013; Liu et al., 2019). Future studies performed in HIV-1-infected cells will be determinant to exclude the possibility that ASP multimerization is a side effect of its overexpression in mammalian cells.

### Subcellular Localization of ASP

Different subcellular localizations were described for ASP *in vitro*, both in transfected cell lines overexpressing ASP (Briquet and Vaquero, 2002; Clerc et al., 2011; Laverdure et al., 2012; Torresilla et al., 2013; Liu et al., 2019) and in HIV-1 infected cell lines expressing endogenous ASP (Briquet and Vaquero, 2002; Affram et al., 2019). Endogenous ASP was localized in the nucleus of PMA-activated chronically infected ACH-2 cells (Briquet and Vaquero, 2002), and was distributed in a nonhomogeneous and polarized manner beneath the nuclear envelope of unstimulated and chronically infected U1C8 T cells (Affram et al., 2019). Within the nucleus of these cells, ASP was detected in areas containing actively transcribed chromatin (Affram et al., 2019). Endogenous ASP was also observed in the cytoplasm of SupT1-infected cells (Briquet and Vaquero, 2002), and PMA-stimulated U1C8 cells (Affram et al., 2019). ASP was also found within the cytoplasm of stably transfected A3.01 T cells (Briquet and Vaquero, 2002) and of transfected COS-7 cells, a simian cell line (Torresilla et al., 2013). In the latter study, ASP was distributed in a punctuate manner within the cytoplasm and was partially co-localized with LC3-IIb, suggesting that it may be associated with autophagosomes (Torresilla et al., 2013). Consistent with both its putative TM domains, ASP was observed at the plasma membranes of PMA-activated, chronically infected U1C8 T cells and myeloid OM 10.1 cells (Affram et al., 2019), of *ex vivo* infected monocyte-derived macrophages and dendritic cells (Laverdure et al., 2012), and also at the plasma membrane of transfected Jurkat cells overexpressing ASP (Clerc et al., 2011). In HIV-1 infected Jurkat cells, ASP was asymmetrically distributed at the plasma membrane (Clerc et al., 2011). Recently, polarized distribution of endogenous ASP at plasma membranes was also observed in chronically infected PMA-activated U1C8 T cells and myeloid OM 10.1 cells, where ASP strongly co-localized with gp120 (Affram et al., 2019). Two experiments indicated that ASP is a *bona fide* component of viral particles: gold-labeled ASP was detected in viral particles released from chronically infected SupT1 cells (Briquet and Vaquero, 2002), and *in vitro* fluorescence correlation spectroscopy (FCS) of cell-free single HIV-1 particles released from PMA-activated U1C8 T-cells suggested that ASP is present at the surface of the viral envelope (Affram et al., 2019).

## ASP and Immune Response

**Figure 5:**
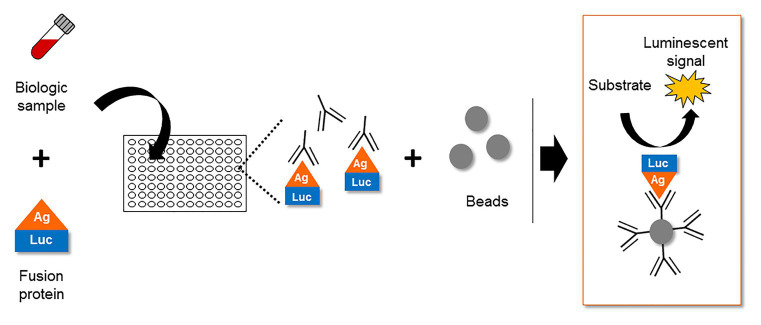
The Luciferase Immuno-Precipitation System (LIPS) as a detection assay to measure antigen-specific antibodies in biological samples of infected patients. The biological samples (plasma, serum, lactoserum, etc.) potentially containing antigen-specific antibodies are incubated with a fusion protein between the antigen and a luciferase. After an immuno-precipitation step, the substrate of the luciferase is added. The detection of a luminescent signal indicates the presence of antigen-specific antibodies in the biological sample.

Despite several *in vitro* reports of ASP in infected and transfected cells, the expression of ASP *in vivo* remained a subject of debate for several years. In this context, the study of the host immune system gave valuable clues to the expression of ASP *in vivo*. The first report of an immune response targeting ASP appeared in 1995, describing the incubation of an *in vitro* translated ASP with the sera of 15 HIV-1 infected individuals (Vanhée-Brossollet et al., 1995). This led to the detection of a band at the expected size of ASP in approximately half of the serum samples by Western blotting (Vanhée-Brossollet et al., 1995). Unfortunately, Western blotting did not allow the frequency of patients displaying antibodies against ASP to be accurately determined. Recently, a quantitative technique known as Luciferase Immunoprecipitation System (LIPS) was described to assess the antibody response targeting ASP in a panel of HIV-1-infected patients (Savoret et al., 2020). LIPS is an assay that was initially developed to quantitatively detect antibodies targeting a particular antigen in patient biological samples (Figure 5; Burbelo et al., 2005). Using LIPS, the breadth of the antibody response directed against different viral antigens, including HIV-1 whole proteome, has previously been reported (Burbelo et al., 2007, 2014; Enose-Akahata et al., 2013; Furuta et al., 2015). Of note, the use of this technique allowed the quantitative study of the antibody response targeting ASP, which was detected in 10–15% of the HIV-1-infected patients (Savoret et al., 2020).

**Figure 6:**
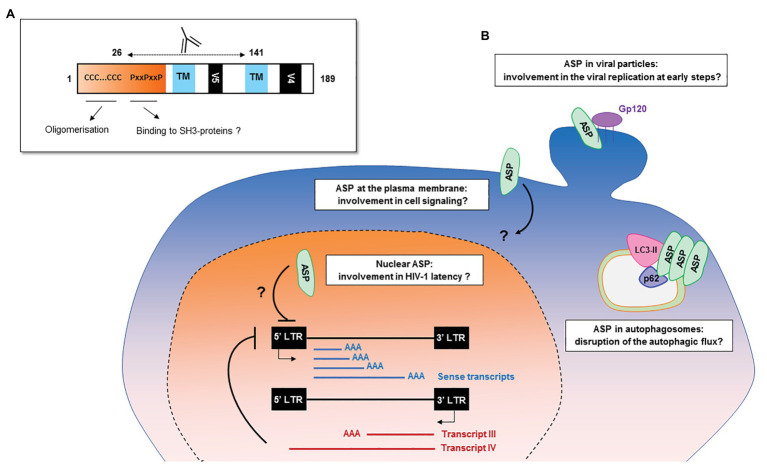
Potential functions of HIV-1 antisense transcripts and ASP in infected cells. **(A)** Potential functions of ASP motifs and localization of its epitope *in vivo*. Patients’ antibodies target the 26–141 core region of ASP encompassing the V5 and proline-rich motifs. **(B)** Schematic representation of the potential functions of ASP and of antisense transcripts in infected cells. In addition to their messenger function, the antisense transcripts of HIV-1 may contribute to the establishment and maintenance of viral latency by recruiting chromatin-modifying enzymes to the 5′ LTR of the proviral genome. ASP oligomers, by interacting with LC3-IIb and p62 in autophagosomes, are degraded by a selective autophagy process and disrupt the autophagic flux of infected cells. At the plasma membrane, ASP could be involved in deregulating infected cells by disrupting cell signaling pathways or the expression of immune receptors. In the nucleus, ASP may be involved in the establishment and maintenance of viral latency, as was described for HBZ. As ASP was detected at the surface of viral particles, it may also play a role in the very early stages of the viral cycle, such as viral replication.

The frequency of patients displaying antibodies to ASP is similar to those observed for both HBZ (Enose-Akahata et al., 2013; Shiohama et al., 2016) and the auxiliary and regulatory proteins of HIV-1 (Reiss et al., 1990; O’Neil et al., 1997; Rezza et al., 2005). The ASP-specific antibody response was sustained for at least 9 months and seemed to target both the 26–62 residues of ASP bearing the highly conserved proline-rich motif and the core residues 62–141 of ASP (Figures 2, 6A; Savoret et al., 2020). Cytotoxic CD8^+^ T cells targeting a panel of ASPs overlapping peptides were also reported in 30% of the HIV-1 infected patients (Berger et al., 2015; Bet et al., 2015). Moreover, the CD8^+^ T cells targeting these peptides produced multiple cytokines and chemokines, indicating that ASP elicited a functional cytotoxic response within patients (Bet et al., 2015).

The abovementioned studies brought strong evidence in favor of the expression of ASP during the course of HIV-1 infection *in vivo* and strongly suggest that ASP elicits an adaptive response in at least some of the HIV-1-infected patients, as previously described for the other auxiliary and regulatory proteins of HIV-1 that elicit both antibodies and CD8^+^ T cell responses (Allan et al., 1985; Arya and Gallo, 1986; Kan et al., 1986; Wong-Staal et al., 1987; Chanda et al., 1988; Strebel et al., 1988; Reiss et al., 1990; O’Neil et al., 1997; Rezza et al., 2005; Kiepiela et al., 2007; Cardinaud et al., 2009; Nicoli et al., 2016). Even though the presence of ASP at the surface of infected cells and viral particles was only reported *in vitro* (Briquet and Vaquero, 2002; Clerc et al., 2011; Laverdure et al., 2012; Affram et al., 2019), it is worth to highlight that an antibody response targeting ASP at the surface of viral particles and infected cells would potentially have interesting implications for the progression of the disease. For future investigations, it would therefore be of great interest to study the functionality of the antibodies targeting ASP *in vivo* and especially their ability to neutralize viral particles or to induce cellular mechanisms contributing to viral clearance such as complement dependent cytotoxicity (CDC), antibody-dependent phagocytosis (ADCP), and antibody-dependent cell cytotoxicity (ADCC), a mechanism whose involvement in the partial success of the RV144 Thai vaccine has been questioned (Kim et al., 2015).

## Functions of Antisense Transcripts and ASP in the Life Cycle of HIV-1

The *asp* gene emerged *de novo* at the beginning of the last century concomitantly with the pandemic group M of HIV-1 (Cassan et al., 2016). The creation of *asp* has several consequences and also questions the evolution of HIV-1. Firstly, *de novo* creation and conservation of *asp* within the HIV-1 M group altered the repertoire of its auxiliary proteins, meaning that HIV-1 acquired an additional factor that is probably not involved in the viral replication cycle *per se*, but which could play a role in pathogenicity or viral transmission (Li and Ding, 2006; Rancurel et al., 2009). Secondly, contrary to the other HIV-1 auxiliary genes, *asp* did not benefit from centuries of evolution, and it may be assumed that it is still evolving. Thirdly, since ASP is a young *de novo* protein, it may not as of yet have adopted a fully compact and specific structure but rather a more rudimentary folding form which may require the intervention of chaperones to partially avoid aggregation and/or use a cellular mechanism like autophagy to degrade aggregated forms (Figure 6). Although the studies of ASP multimerization and its link with autophagy were performed in cell lines overexpressing ASP (Torresilla et al., 2013; Liu et al., 2019) and would need to be confirmed by future studies in HIV-1-infected cells, it is tempting to speculate that one pool of ASP could fold into a monomeric functional form in infected cells, while another pool folds into oligomers of non-functional forms. Conversely, we can also speculate that ASP forms non-toxic and functional oligomers that would represent an additional way to subvert the autophagic flux of the cell to the benefit of HIV-1.

On the one hand, it is thus possible that the oligomerization of ASP mediates its own degradation by a process of selective autophagy while at the same time disrupting the autophagic flux of infected cells, as has been previously described for the viral proteins Tat, Nef, and Vif (Borel et al., 2015; Sagnier et al., 2015; Sardo et al., 2015; Saribas et al., 2015). As subtype-A ASP is devoid of the N-terminal part required for ASP oligomerization (Liu et al., 2019), it could be of interest to wonder whether ASP from subtype A represents a form of ASP which evolved to avoid ASP multimerization and to partially counteract its own degradation by autophagy. On the other hand, the fact that *asp* did not benefit from centuries of evolution to adapt the expression of ASP in host cells could argue in favor of the hypothesis stipulating that ASP multimers are deleterious both for the cell and the virus.

Different hypotheses relating to the functions endorsed by ASP can be inferred from its different subcellular localizations. Like HBZ, a key protein in the establishment and maintenance of HTLV-1 latency (Gaudray et al., 2002; Barbeau and Mesnard, 2015; Matsuoka and Mesnard, 2020), nuclear ASP could contribute to viral latency by interacting with proteins involved in the regulation of gene expression, for example, chromatin modifying enzymes or transcription factors. In addition to encoding ASP, antisense transcripts may also, as previously discussed, exert a role in the establishment and maintenance of viral latency (Figure 6; Kobayashi-Ishihara et al., 2012, 2018; Saayman et al., 2014; Zapata et al., 2017).

It is possible that at the plasma membrane of infected cells, ASP could modulate the expression of immune receptors as previously described for other HIV-1 auxiliary proteins (Guy et al., 1987; Schwartz et al., 1996; Stumptner-Cuvelette et al., 2001; Sol-Foulon et al., 2002; Hrecka et al., 2005; Michel et al., 2005; Chaudhry et al., 2009; Sauter et al., 2009; Shah et al., 2010) or interfere with cell signaling pathways by interacting with SH3-proteins through its PxxP motifs (Figure 6). ASP might also favor optimal HIV-1 replication at early steps of HIV-1 cycle. Indeed, HEK 293 T cells transfected with a pNL4.3 construction carrying an abortive mutation in the sequence encoding ASP showed lower extracellular p24 levels than cells transfected with wild type pNL4.3 (Clerc et al., 2011). Corroborating results were obtained in HEK 293 T cells, HeLa cells, and U937 monocytic cells transfected with a differently ASP-mutated version of pNL4.3, as well as in U937 cells infected with VSVg-pseudotyped ASP-mutated pNL4.3 virions: ASP-mutated pNL4.3 led to reduced p24 extracellular levels compared to wild type pNL4.3 (Torresilla et al., 2013). The detection of ASP at the surface of HIV-1 particles released from infected cells may also indicate that ASP exerts an effect at an early stage of the viral cycle (Briquet and Vaquero, 2002; Affram et al., 2019).

It may be speculated that ASP could perform different functions according to the stage of the viral cycle: It might promote viral replication at an early stage, and conversely, it might promote the maintenance of viral latency once established. Furthermore, ASP could possibly be involved in the deregulation of infected cells by disrupting (i) the autophagic flux, (ii) cell signaling-pathways, or (iii) the expression of immune receptors (Figure 6). Like other HIV-1 auxiliary proteins, ASP is therefore probably a pleiotropic protein. The exploration of ASP presents a particular challenge, and unraveling its potential functions will require the combined study of productively and latently infected cells, as well as the development or optimization of tools and techniques to detect this hydrophobic protein which might be expressed in very low amounts by infected cells.

## Functions of Antisense Transcripts and Antisense Proteins in Other Viruses

As we saw above, antisense transcription is not exclusive to HIV-1. Indeed, not only some other lentiviruses (FIV, BIV), deltaretroviruses (HTLV-1, HTLV-2, HTLV-3, HTLV-4, STLV-1,and BLV), and gammaretroviruses (MLV) but notably some phylogenetic divergent viruses as *Herpesviridae* are capable of so-called antisense transcription (Spivack and Fraser, 1987; Larocca et al., 1989; Flemington and Speck, 1990; Cheung, 1991; Holden et al., 1992; Prang et al., 1995; Perng et al., 1996, 2002; Speck et al., 1997; Jiang et al., 1998; Borchers et al., 1999; Ciacci-Zanella et al., 1999; Briquet et al., 2001; Gaudray et al., 2002; Inman et al., 2004; Rajčáni et al., 2004; Bego et al., 2005; Calattini et al., 2005, 2006; Jones et al., 2006; Ahn et al., 2007, 2010; Ou et al., 2007; Duellman et al., 2009; Halin et al., 2009; Rasmussen et al., 2010; Larocque et al., 2011, 2014; Barbeau et al., 2013; Miura et al., 2013; Barbeau and Mesnard, 2015; Barez et al., 2015; Daskalogianni et al., 2015; Liu et al., 2015; Durkin et al., 2016; Fochi et al., 2018; Moldován et al., 2018; Harrod, 2019; Jégado et al., 2019; Martinez et al., 2019; Tagaya et al., 2019; Hau et al., 2020; Matsuoka and Mesnard, 2020). In cell lines infected with laboratory-adapted FIV isolates and in various lymphoid tissues of cats infected by a FIV primary isolate, antisense transcripts arising from an antisense ORF that is complementary to the FIV *env* gene were detected (Briquet et al., 2001). Interestingly, the antisense ORF was shown to be conserved in five FIV isolates (Briquet et al., 2001). Antisense transcripts were also detected in HEK 293 T cells transfected with the BIV-127 proviral clone and in BIV-permissive cell lines infected with BIV-127 (Liu et al., 2015).

### Antisense Transcripts and Antisense Proteins in Retroviruses

HTLVs are composed of four members: HTLV-1, which is the etiological agent of adult T-cell leukemia/lymphoma (ATLL) and HTLV-1 associated myelopathy/tropical spastic paraparesis (HAM/TSP); HTLV-2, for which no clinical correlation with HAM/TSP or lymphoproliferative disease has been established, though it was initially discovered in a patient displaying a rare benign form of hairy T-cell leukemia; HTLV-3, which might present some transforming abilities; and HTLV-4, for which very few clinical data are available (Roucoux and Murphy, 2004; Duong et al., 2008; Chevalier et al., 2012; Tagaya et al., 2019). HTLV-1, HTLV-2, HTLV-3, and HTLV-4 are complex retroviruses that encode several regulatory and auxiliary products. The viral transcription of the HTLVs is stimulated by a complex composed of the transactivator of pX (Tax) and transcription factors such as the cAMP response element binding protein (CREB) but also histone acetyl transferases CBP/p300, which binds to sequences called cAMP response element (CRE) within the retroviral promoter.

Human T-cell leukemia virus type 1 encodes Tax-1, a transforming protein *in vivo* that activates not only the viral sense transcription from the 5’LTR but also many host genes through the activation of the NF-*κ*B and CREB/ATF pathways. HTLV-2, HTLV-3, and HTLV-4 respectively, encode Tax-2, which displays transforming abilities *in vitro*, Tax-3, and Tax-4 transactivators (Calattini et al., 2006; Chevalier et al., 2012; Gessain et al., 2013; Shirinian et al., 2013). The protein derived from the antisense transcripts produced from the 3′ LTR of HTLV-1, called HBZ, together with the *hbz* mRNA, has shown to play a crucial role in HTLV-1 replication and its associated pathologies (ATLL, HAM/TSP; Gaudray et al., 2002; Satou et al., 2006; Barbeau et al., 2013; Barbeau and Mesnard, 2015; Tagaya et al., 2019; Matsuoka and Mesnard, 2020). Indeed, the *hbz* mRNA promotes the proliferation of ATL cells while HBZ plays a central role in the process of oncogenesis and is interfering with many cellular processes (innate immune signaling, apoptosis, autophagy, DNA repair, and genes expression; Mitobe et al., 2015; Zhao, 2016; Tagaya et al., 2019; Matsuoka and Mesnard, 2020).

The HTLV-2, HTLV-3, and HTLV-4 nonconventional basic zipper (bZIP) proteins that are encoded by antisense transcripts are named ASP of HTLV-2/3/4 (APH-2, APH-3, and APH-4, respectively), and are functional synologs of HBZ (Halin et al., 2009; Larocque et al., 2011). HBZ has been shown to interact *via* its bZIP domain with several cellular transcription factors such as CREB, CREB2, JunD, c-Jun, JunB, and the p65 subunit of the NF-B complex. By heterodimerizing with CREB, HBZ is preventing its recruitment to the 5’LTR and, therefore, inhibiting HTLV-1 sense transcription, which facilitates the entry of HTLV-1-infected cells into latency (Matsuoka and Mesnard, 2020). Conversely, several studies showed that HBZ could have a positive impact on its own expression by stimulating the transcription from the 3’LTR through the binding of a complex including JunD and Sp1 on the Sp1 binding sites located in the U5 region of the 3' LTR (Satou et al., 2006; Gazon et al., 2012). Altogether, HBZ plays an essential role in the regulation of HTLV-1 expression by acting as a negative regulator of the viral sense transcription, which in turns inhibit Tax-1 expression and viral particle production, and by positively modulating the viral antisense transcription and thus its own expression (Barbeau and Mesnard, 2015; Tagaya et al., 2019; Matsuoka and Mesnard, 2020).

Like HBZ, APH-2 can interact with CREB and thus inhibit the transactivation of HTLV-2 sense transcription. However, APH-2 does not possess a classical bZIP domain and interacts with CREB through a leucine-rich pattern LXXLL. Unlike HBZ, APH-2 possesses the ability to interact directly with Tax-2 to inhibit the Tax/CREB-dependent sense transcription and to positively regulate JunB and c-Jun through an interaction involving its nonconventional bZIP domain (Barbeau and Mesnard, 2015; Fochi et al., 2018; Harrod, 2019; Martinez et al., 2019). Although only few studies have been performed on APH-3 and APH-4, these antisense proteins display common features with HBZ and APH-2, suggesting that they might play an important role in HTLV-3 and HTLV-4 infections. Like APH-2, but unlike HBZ, APH-3 and APH-4 are able to activate c-Jun, JunB, and JunD through the interaction of their nonconventional bZIP domain with these factors. Although some of the functions of APH-2, APH-3, APH-4, and HBZ are divergent, APH-3 and APH-4 share with HBZ and APH-2 the ability to inhibit retroviral sense transcription (Larocque et al., 2011, 2014).

In 2013, STLV-1 spliced transcripts corresponding to HTLV-1 *tax*, and HTLV-1 *hbz* transcripts were identified in STLV-1-infected cells from naturally infected Japanese macaques (Miura et al., 2013). The products of these transcripts, named Tax and SBZ, seem to share similar functions with their HTLV-1 counterparts (Miura et al., 2013). The identification of BLV microRNAs and the recent identification of BLV antisense transcripts represent a major shift in the understanding of BLV pathogenesis (Kincaid et al., 2012; Rosewick et al., 2013; Durkin et al., 2016). Indeed, in contrast to the previously prevailing paradigm of a silent BLV provirus, these discoveries show that BLV provirus is producing viral microRNAs and antisense transcripts in all the tumors that were investigated. The consistent expression of these antisense transcripts in both leukemic and nonmalignant cells suggests that they are playing a crucial role in the virus life cycle and its tumorigenic potential (Durkin et al., 2016). Besides, it has been shown that MLV can initiate transcription from the U3 region of the negative strand of its proviral genome to produce transcripts of negative polarity (Rasmussen et al., 2010).

All these results suggest that antisense transcription might be a rule rather than an exception in retroviruses. In this context, the study of the role exerted by antisense transcripts and potential/proven ASPs in other retroviruses, as well as in endogenous retroviruses, could be of particular interest to enhance our understanding of the impact of these retroviruses on human biology and on numerous pathologies (Manghera et al., 2017). However, antisense transcription is not limited to retroviruses and has also been described in numerous other viruses, such as herpesviruses (HVs).

### Antisense Transcripts and Antisense Proteins in Herpesviruses

Antisense transcription has also been particularly studied in the *Herpesviridae* family. HVs are double-stranded DNA viruses which possess large genomes that encode hundreds of proteins. HV infections can remain unnoticed or on the contrary be associated with a wide range of pathologies in their natural host (Mahalingam and Gilden, 2007; Griffiths et al., 2015; Hammerschmidt, 2015; Yildirim et al., 2015; Kennedy and Gershon, 2018; Jones, 2019; Saleh and Sharma, 2020; Sehl and Teifke, 2020). HV infections lead to persistent infections that are characterized by latency and lytic phases. Interestingly, the use of sense or antisense transcription within HVs seems to be strongly associated with these two distinct phases of infection (Rovnak et al., 2015; Phelan et al., 2017).

The molecular mechanisms underlying viral latency/reactivation of HVs have been the subject of numerous studies. In the case of HSV-1, both types of infection can be developed through the coexistence in the viral genome of two alternative gene expression programs that are notably under the control of epigenetic mechanisms (Rovnak et al., 2015). HSV-1 genome possesses a Unique Long (UL) region, which is flanked by the identical but inverted Repeat Longs (RLs), and a Unique Short (US) region, which is flanked by the identical but inverted Repeat Shorts (RS). During the lytic phase, a ternary complex, including the viral tegument protein VP16/host cell factor 1 (HCF1)/octamer binding protein 1 (OCT1), associated with CBP/p300 and lysine demethylases (LSD1), interacts with the promoters of very early viral genes, preventing the formation of repressive heterochromatin and activating the expression of very early proteins: ICP0, ICP4, ICP22, ICP27, and ICP47. These proteins then regulate the expression of early genes that are coding for proteins involved in DNA replication, with the exception of ICP47, which assists the virus in avoiding the host immune response, and late genes that are coding for capsid proteins, the tegument envelope, and the viral envelope. During latent infection, HSV-1 lytic genes are silenced and the only HSV-1 transcripts that are detected are named Latency Associated Transcripts (LATs) and map the RL (Stevens et al., 1988; Phelan et al., 2017). Among these transcripts, the first to be described was a RNA of 8.3 kb that is antisense to the ICP0 and ICP34 encoding genes. Later, spliced products, called “major LATs” of 2.0 kb and 1.5, were also characterized (Phelan et al., 2017). However, more recently, it has been shown that the region encoding the LATs is transcriptionally much more complex than originally described (Stevens et al., 1988; Rovnak et al., 2015; Phelan et al., 2017). Indeed, this region encodes also several additional noncoding RNAs and about a dozen miRNAs. A number of phenotypes were found to be associated with the expression of the LAT genes, and by extension to the LATs, including establishment and reactivation from latency, apoptosis regulation, neuronal survival, and modulation of the innate immunity (Du et al., 2011, 2012, 2013; Phelan et al., 2017; Watson et al., 2018; Barrozo et al., 2020a,b). Through the viral life cycle, the epigenetic profile of the HSV-1 genome was shown to change in a LATs-dependent manner. Indeed, during the transition into latency phase, repressive histone modifications (methylated H3K9) accumulate on HSV-1 promoters, and functional LAT genes are associated with more abundant heterochromatin. Interestingly, LAT-dependent heterochromatin modification on lytic virus promoters was found to be linked to modifications of host PRC-2 (Kwiatkowski et al., 2009; Cliffe et al., 2013).

Antisense transcription has also been described for other species within the family of the Herpesviridae. Indeed, EBV (Flemington and Speck, 1990; Prang et al., 1995; Speck et al., 1997; Duellman et al., 2009; Daskalogianni et al., 2015; Hau et al., 2020) encodes an oncoprotein named Zta (BZLF1, ZEBRA, EB1), which is a bZIP transcription factor and a key regulator of the switch from latent to lytic phases of the virus life cycle (Lee et al., 1987; Lieberman and Berk, 1990; Sinclair, 2003; Bhende et al., 2004; Kenney, 2007; Tsai et al., 2009; Ramasubramanyan et al., 2012; Gustems et al., 2014; Hong et al., 2017; Germini et al., 2020). Antisense transcription has also been detected in HCMV (Ma et al., 2012), VZV (Markus et al., 2017; Depledge et al., 2018; Bisht et al., 2020), SVV (Ou et al., 2007), EHV1 and EHV4 (Holden et al., 1992; Borchers et al., 1999; Ahn et al., 2007), pseudorabies virus (PRV; Jiang et al., 1998; Ciacci-Zanella et al., 1999; Inman et al., 2004; Jones et al., 2006; Wu et al., 2012; Klionsky et al., 2016; Wang et al., 2018), and BHV-1 (Jiang et al., 1998; Ciacci-Zanella et al., 1999; Inman et al., 2004; Jones et al., 2006; Glazov et al., 2010).

Altogether, antisense transcription in viruses appears more widespread than expected and could highlight an evolutionary and functional convergence between families of viruses that are phylogenetically distant. Indeed, as we saw previously, the antisense proteins and/or the antisense transcripts may be endorsed with important functions in viral infections, including the control of viral sense transcription and viral latency. Beyond that, viral antisense actors may be essential to maintain a latent reservoir and to modulate virulence, which may in turn confer a tremendous selective advantage for the virus by maintaining a longer lasting source of spreading infection. Although several proposals have been made concerning the function of ASP and antisense transcripts in HIV-1 infection, the exact roles endorsed by these antisense actors in HIV-1 replication cycle and physiopathology still remain to be defined (Clerc et al., 2011; Kobayashi-Ishihara et al., 2012; Laverdure et al., 2012; Torresilla et al., 2013; Saayman et al., 2014; Zapata et al., 2017; Affram et al., 2019; Liu et al., 2019).

## Discussion and Perspectives

HIV-1 has the typical retrovirus genomic organization, and contains both regulatory and auxiliary genes. Until recently, retroviral antisense transcription was not evaluated or even considered as a new source of viral transcripts and proteins playing important roles in the viral life cycle. However, this viewpoint has evolved with accumulating evidence of antisense transcription in several retroviruses, and the discovery of HBZ plays different roles in the pathogenesis of HTLV-mediated T-cell leukemia (Gaudray et al., 2002; Barbeau and Mesnard, 2015; Matsuoka and Mesnard, 2020). The ability of HIV-1 to produce antisense transcripts and ASP *in vitro* has been well established; many studies have described the expression of antisense transcripts and ASP in various HIV-1 infected cells, including T-cells, monocyte-derived macrophages, and dendritic cells (Ludwig et al., 2006; Landry et al., 2007; Clerc et al., 2011; Kobayashi-Ishihara et al., 2012; Torresilla et al., 2013; Affram et al., 2019). Moreover, CD8^+^ T cells and antibodies targeting ASP were detected in HIV-1 infected patients (Vanhée-Brossollet et al., 1995; Berger et al., 2015; Bet et al., 2015; Savoret et al., 2020). Bioinformatic approaches demonstrated that a conserved *asp* gene was created, concomitant with the emergence of the HIV-1 pandemic group M (Cassan et al., 2016), further supporting the idea that the *asp* gene might now be considered as the 10th gene of HIV-1. The presence of the overlapping *asp* gene in the −2 frame of the *env* gene shows that HIV-1 has evolved to increase its coding capacity but, at the same time, has also increased the level of constraints imposed by overlapping genes.

The *de novo* creation of an overlapping gene on the antisense strand of the *env* gene, the expression of the “young” and pleiotropic protein ASP, together with the potential expression of various viral antisense transcripts possibly involved in viral latency, support the fact that HIV-1 is a complex retrovirus and provide new evidence of the HIV-1 evolution process. Future research avenues will be directed to understand the precise functions of these new elements during the course of HIV-1 infection. The potential role of ASP and its transcripts in viral replication and latency, the ability of ASP to elicit both arms of adaptive immunity, and its potential expression at the surface of infected cells and viral particles could make ASP an interesting new target for antiretroviral treatment and vaccine strategies.

## Author Contributions

Conceptualization of the article and writing of the original draft was done by JS and NC. Writing, reviewing, and editing the final version was done by J-MM and AG. All authors contributed to the article and approved the submitted version.

### Conflict of Interest

The authors declare that the research was conducted in the absence of any commercial or financial relationships that could be construed as a potential conflict of interest.

## Glossary

**Table:**

Term	Definition
ADCC	Antibody dependent cell cytotoxicity
ADCP	Antibody dependent phagocytosis
APH-2/3/4	Antisense protein of HTLV-2/3/4
ASP	Antisense protein
ATLL	Adult T cell leukemia/lymphoma
BHV-1	Bovine herpes virus type-1
BIV	Bovine immunodeficiency virus
BLV	Bovine leukemia virus
CDC	Complement dependent cytotoxicity
CRE	cAMP response element
CREB	cAMP response element binding protein
CRF	Circulating recombinant form
DNMT3a	DNA methyltransferase 3a
EGFP	Enhanced green fluorescent protein
EHV1/4	Equine herpesvirus 1 and 4
EZH2	Enhancer of zeste homolog 2
FCS	Fluorescence correlation spectroscopy
FIV	Feline immunodeficiency virus
HAM/TSP	HTLV-1 associated myelopathy/topical spastic paraparesis
HBZ	HTLV-1 Basic leucine zipper factor
HCF-1	Host cell factor 1
HCMV	Human cytomegalovirus
HDAC	Histone deacetylase
HIV-1	Human immunodeficiency virus type 1
HTLV-1/2/3/4	Human T-cell leukemia virus type-1/2/3/4
HV	Herpesvirus
ICP	Infected cell protein
LAT	Latency associated transcripts
LC3	Microtubule-associated protein light chain 3
LIPS	Luciferase immuno-precipitation system
Lnc	Long non-coding
LSD1	Lysine demethylase
LTR	Long terminal repeat
MLV	Murine leukemia virus
OCT1	Octamer binding protein 1
ORF	Open reading frame
PBMC	Peripherical blood mononuclear cells
PHA	Phytohaemagglutinin
PMA	Phorbol myristate acetate
PRC2	Polycomb repressor complex 2
PRV	Pseudorabies virus
RL	Repeat longs
RRE	Rev responsive element
RS	Repeat shorts
SBZ	STLV-1 basic leucine zipper factor
STLV-1	Simian T-Leukemia virus type 1
SVV	Simian varicella virus
UL	Unique long
US	Unique short
V4/V5	Hypervariable region V4 and V5
VZV	Varicella-zoster virus
